# Phoyunnanin E inhibits migration of non-small cell lung cancer cells via suppression of epithelial-to-mesenchymal transition and integrin αv and integrin β3

**DOI:** 10.1186/s12906-017-2059-7

**Published:** 2017-12-29

**Authors:** Nareerat Petpiroon, Boonchoo Sritularak, Pithi Chanvorachote

**Affiliations:** 1 Cell-Based Drug and Health Product Development Research Unit, Faculty of Pharmaceutical Sciences, Chulalongkorn University, Bangkok, 10330 Thailand; 2 Department of Pharmacology and Physiology, Faculty of Pharmaceutical Sciences, Chulalongkorn University, Bangkok, 10330 Thailand; 3 Department of Pharmacognosy and Pharmaceutical Botany, Faculty of Pharmaceutical Sciences, Chulalongkorn University, Bangkok, 10330 Thailand

**Keywords:** Phoyunnanin E, Migration, Lung cancer, Integrin, Epithelial to mesenchymal transition

## Abstract

**Background:**

The conversion of the epithelial phenotype of cancer cells into cells with a mesenchymal phenotype-so-called epithelial–mesenchymal transition (EMT)-has been shown to enhance the capacity of the cells to disseminate throughout the body. EMT is therefore becoming a potential target for anti-cancer drug discovery. Here, we showed that phoyunnanin E, a compound isolated from *Dendrobium venustum,* possesses anti-migration activity and addressed its mechanism of action.

**Methods:**

The cytotoxic and proliferative effects of phoyunnanin E on human non-small cell lung cancer-derived H460, H292, and A549 cells and human keratinocyte HaCaT cells were investigated by MTT assay. The effect of phoyunnanin E on EMT was evaluated by determining the colony formation and EMT markers. The migration and invasion of H460, H292, A549 and HaCaT cells was evaluated by wound healing assay and transwell invasion assay, respectively. EMT markers, integrins and migration-associated proteins were examined by western blot analysis.

**Results:**

Phoyunnanin E at the concentrations of 5 and 10 μM, which are non-toxic to H460, H292, A549 and HaCaT cells showed good potential to inhibit the migratory activity of three types of human lung cancer cells. The anti-migration effect of phoyunnanin E was shown to relate to the suppressed EMT phenotypes, including growth in anchorage-independent condition, cell motility, and EMT-specific protein markers (N-cadherin, vimentin, slug, and snail). In addition to EMT suppression, we found that phoyunnanin E treatment with 5 and 10 μM could decrease the cellular level of integrin αv and integrin β3, these integrins are frequently up-regulated in highly metastatic tumor cells. We further characterized the regulatory proteins in cell migration and found that the cells treated with phoyunnanin E exhibited a significantly lower level of phosphorylated focal adhesion kinase (p-FAK) and phosphorylated ATP-dependent tyrosine kinase (p-AKT), and their downstream effectors (including Ras-related C3 botulinum (Rac-GTP); Cell division cycle 42 (Cdc42); and Ras homolog gene family, member A (Rho-GTP)) in comparison to those of the non-treated control.

**Conclusions:**

We have determined for the first time that phoyunnanin E could inhibit the motility of lung cancer cells via the suppression of EMT and metastasis-related integrins. This new information could support further development of this compound for anti-metastasis approaches.

## Background

Metastasis has long been recognized as one of the important causes of the high death rate in lung cancer cases worldwide [1]. The key molecular processes that facilitate the metastasis potential of cancer cells have been intensively investigated and the process of cancer cell alteration from epithelial forms to more motile phenotypes, known as epithelial to mesenchymal transition (EMT), has long been shown to play a principle role in supporting metastasis. EMT increases cell migration, invasion and survival in anchorage-independent conditions [2–4], which are the primary properties of cancer cells that should succeed in metastasis. The critical hallmarks of EMT are the E-cadherin to N-cadherin switch, and the up-regulation of vimentin, slug and snail proteins [5–7]. Likewise, current research has pointed out that the function and cellular levels of certain integrins, including αv and β3, can facilitate metastasis in many cancers, including lung cancer [8–10]. The up-regulation of αv and β3 integrins has been shown to be predominantly found in metastatic cancers [11–13]. Integrins play a key role as a receptor on the cell side in binding with extracellular-matrix ligands and providing survival signals for the attached cells [14–16]. Normally, integrins consist of alpha and beta subunits, and initiate transmembrane signaling by activating focal adhesion kinase (FAK) and further activate various downstream effectors, such as Protein kinase B (AKT) and the Ras homolog gene family (Rho family) (Cell division cycle 42 (Cdc42), Ras-related C3 botulinum toxin substrate 1 (Rac1), and Ras homolog gene family, member A (RhoA) GTPases), for cancer metastasis [4, 17, 18]. Therefore, alterations of these molecules could competently prevent cancer metastasis.

**Fig. 1:**
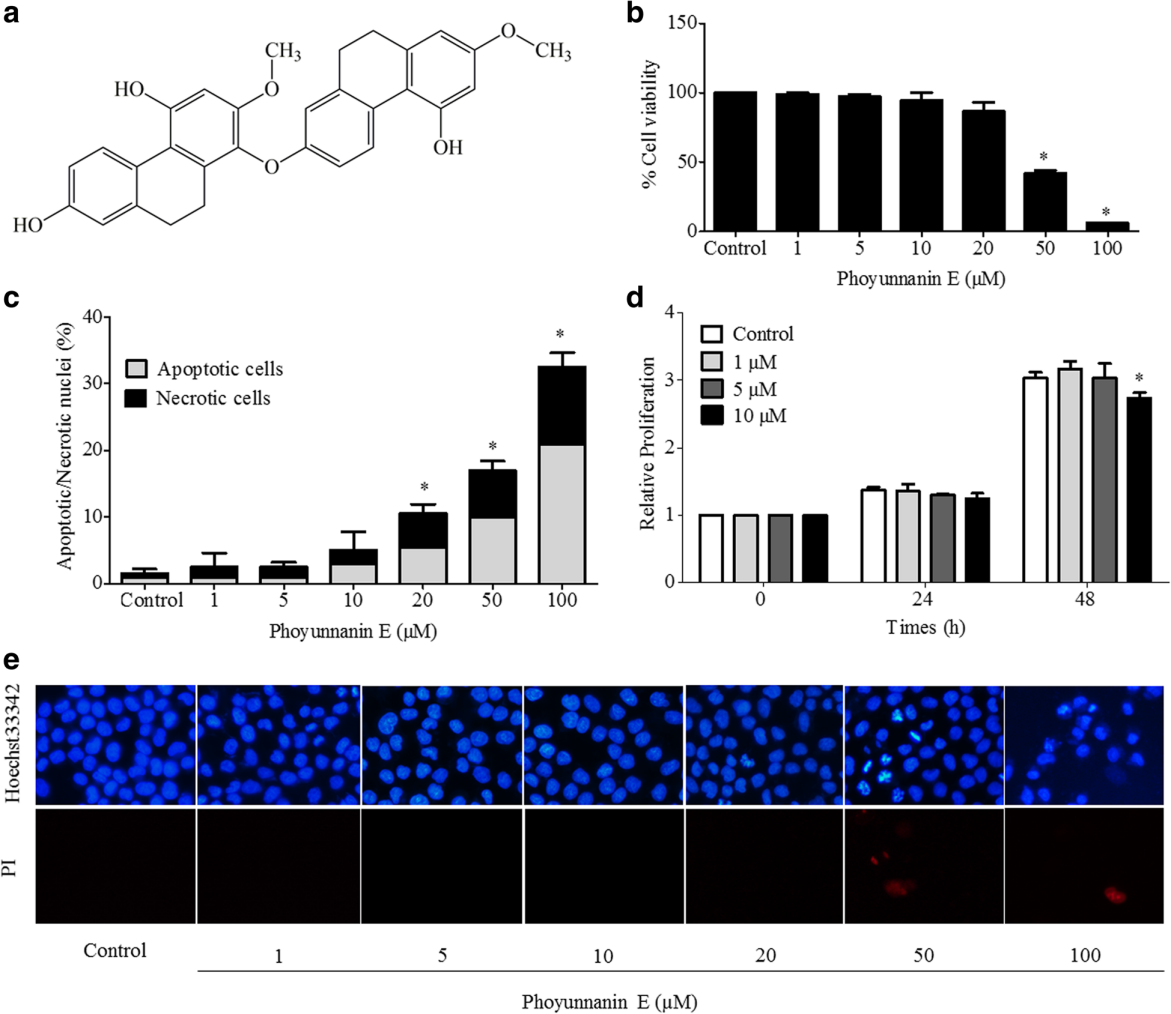
Structure of Phoyunnanin E (**a**). Viability of non-small cell lung cancer cells (H460) in response to various concentrations of phoyunnanin E (0–100 μM) treatment for 24 h (**b**). Cell viability was evaluated using 3-[4,5-dimethylthiazol-2-yl]-2,5 diphenyl tetrazoliumbromide (MTT) assays. Percentages of apoptotic and necrotic nuclei in cells treated with phoyunnanin E (**c**). Apoptotic and necrotic cell death after phoyunnanin E treatment, determined by Hoechst 33342/PI co-staining and visualized by fluorescence microscopy (**e**). Proliferation of the cells after treatment with phoyunnanin E, at 24 and 48 h (**d**). Data are shown as the mean ± SD (*n* = 3). * *P* < 0.05 versus non-treated control

*Dendrobium venustum* Teijsm. & Binn. (Orchidaceae) is found in the north, northeast, central and west of Thailand. It known in Thai as “Ueang Dok Ma Kham” [19]. In a previous study, several phenolic compounds have been isolated from the whole plant of this plant which include flavanthrinin, gigantol, densiflorol B, lusianthridin, batatasin III, phoyunnanin E, and phoyunnanin C. Phoyunnanin E and densiflorol B exhibited strong antimalarial activity [20]. However, the effect of phoyunnanin E on cancer therapeutics has not been investigated. Therefore, the present study aimed to investigate the effects of phoyunnanin E (Fig. 1), a pure compound isolated from *D. venustum,* on key metastasis-related pathways in human lung cancer cells. The researcher also extended this work to cover the consequent effects of the compound on anchorage-independent growth, metatstasis-related integrins, and downstream migratory effectors. The results from this study may benefit the development of this compound for anti-metastasis therapy.

## Methods

### Phoyunnanin E preparation

Phoyunnanin E was isolated from D. *venustum*. as previously described [20]. *D. venustum* was purchased from Jatujak market, Bangkok, in May 2012. Authentication was performed by comparison with herbarium specimens at the Department of National Park, Wildlife and Plant Conservation, Ministry of National Resources and Environment. A voucher specimen (BS-DV-052555) was deposited at the Department of Pharmacognosy, Faculty of Pharmaceutical Sciences, Chulalongkorn University, Bangkok, Thailand. The dried and powdered whole plant (2 kg) was macerated with MeOH (3 × 10 L) to afford a MeOH extract (164 g) after removal of the solvent. This material was subjected to vacuum-liquid chromatography on silica gel (n-hexane EtOAc gradient) to give 8 fractions (A-H). Fraction G (16.3 g) was fractionated by column chromatography over silica gel eluting with a CH2Cl2-EtOAc gradient to give 10 fractions (GI-GX). Phoyunnanin E (16 mg), was obtained in Fraction GVII (2.2 g). Its purity was determined using NMR spectroscopy. Phoyunnanin E with more than 95% purity was used in this study.

### Cells and reagents

Human non-small cell lung cancer (NSCLC)-derived H460, H292, A549 cells were purchased from the American Type Culture Collection (Manassas, VA, USA). The human keratinocyte (HaCaT) cells were purchased from Cell Lines Service (Heidelberg, Germany). H460 and H292 cells were cultured in Roswell Park Memorial Institute (RPMI) 1640 medium (Gibco, Grand Island, NY, USA). A549 and HaCaT were cultured in Dulbecco’s Modified Eagle’s Medium (DMEM, Gibco, Grand Island, NY, USA). The medium was supplemented with 10% fetal bovine serum (FBS), 2 mM L-glutamine and 100 units/ml of each penicillin and streptomycin (Gibco, MD, USA) at 37 °C with 5% CO_2_ in a humidified incubator. Phoyunnanin E was isolated from *D. venustum* and dissolved in dimethylsulfoxide (DMSO) at the indicated working concentrations. The amount of DMSO in the final solution was less than 0.5%, which produced no cytotoxicity in H460 cells. 3-(4,5-dimethylthiazol-2-yl)-2,5-diphenyltetrazoliumbromide (MTT), DMSO, Hoechst33342, propidium iodide (PI), Phalloidin-Rhodamine and bovine serum albumin (BSA) were purchased from Sigma Chemical, Inc. (St. Louis, MO, USA). Antibodies directed against N-cadherin, E-cadherin, vimentin, snail, slug, integrin αv, α5, β3, Focal Adhesion Kinase (FAK), phosphorylation of Focal Adhesion Kinase on Try397 (p-FAK (Try397)), Protein Kinase B (Akt), phosphorylation of Protein Kinase B on Ser473 (p-Akt (Ser473)), Cell division cycle 42 (Cdc42), active Ras-related C3 botulinum toxin substrate 1 (RacGTP) and Glyceraldehyde 3-phosphate dehydrogenase (GAPDH), and its respective secondary antibodies were purchased from Cell Signaling (Danvers, MA, USA). Antibodies directed against active Ras homolog gene family, member A (Rho-GTP) was purchased from NewEast Bioscience (King of Prussia, PA, USA).

### Cytotoxicity assay

Cell viability was determined by MTT assay, as previously described [5]. H460, H292, A549 and HaCaT cells were seeded at a density of 1 × 10^4^ cells/well in 96-well plates and treated with phoyunnanin E (0–100 μM) for 24 h at 37 °C. After treatment, the cells were incubated with MTT (0.4 mg/ml) for 4 h at 37 °C. Then, the supernatant solution was removed and 100 μl DMSO was added to dissolve the crystal formazan product. The resulting formazan intensities were measured by spectrophotometry at 570 nm using a microplate reader (Anthros, Durham, NC, USA). The percentage of cell viability was calculated as absorbance of phoyunnanin E treated cells relative to non-treated cells.

### Proliferation assay

Cell proliferation was analyzed using a colorimetric MTT assay, as previously described [21]. H460 cells were seeded at a density of 2 × 10^3^ cells/well in 96-well plates and treated with non-toxic concentrations of phoyunnanin E (0–10 μM) for 0, 24 and 48 h at 37 °C. Next, cell proliferation was determined through incubation with MTT (0.4 mg/ml) for 4 h at 37 °C, after which the optical densities (OD) of the formazan products were measured at 570 nm using a microplate reader (Anthros, Durham, NC, USA). The proliferation rates were determined using the following equation: OD at indicated time / OD at time 0. The relative proliferation rates were determined by comparing the proliferation rates of treated cells with those of untreated control cells.

### Nuclear staining assay

H460, H292, A549 and HaCaT cells were seeded in 96-well plates at a density of 1 × 10^4^ cells/well, incubated overnight, and treated with phoyunnanin E at various concentration (0–100 μM) for 24 h at 37 °C. Next, the cells were incubated with 10 μg/ml Hoechst 33342 and 5 μg/ml propidium iodide (PI) for 30 min at 37 °C, as previously described [5]. The apoptotic cells having condensed chromatin and fragmented nuclei and PI-positive nerotic cells were captured by using a fluorescence microscope (Olympus IX5; 40×) equipped with a DP70 digital camera system (Olympus, Tokyo, Japan).

### Anchorage-independent growth assay

Anchorage-independent growth was determined by the soft agar colony-formation assay, modified from Phiboonchaiyanan PP. et al.’s method [22]. H460 cells were pre-treated with phoyunnanin E at non-toxic concentrations (0–10 μM) for 48 h at 37 °C. Soft agar was prepared by using a 1:1 mixture of RPMI 1640 medium containing 10% FBS and 1% agarose, and then 500 μl of the mixture was put in a 24-well plate and allowed to solidify at 4 °C for 15 min. To prepare the upper layer, it consisting of 3 × 10^3^ cells/mL in the agarose gel with 10% FBS and 0.3% agarose was added. After the upper layer solidified, the cultured medium was added over the upper layer and incubated at 37 °C for two weeks. Fresh culture medium (200 μl/well) was fed to the system every three days. Colony formation was determined using a phase-contrast microscope (Olympus 1×51 with DP70). The percentage of colony number and diameter were calculated using the following formula: (number of colony or diameter of the phoyunnanin E-treated cells × 100) / number of colony or diameter of control.

### Wound healing assay

Wound healing assay used for detecting cell migration, as previously described [21]. Next, the monolayer cells were allowed to migrate after scratching the attached cells to generate a wound space with a P200 micropipette tip. Detached cells were removed by rinsing once with PBS, after which RPMI1640 medium with 1% FBS was added. At indicated time points (0, 24 and 48 h), phase contrast images of the wound spaces were captured under a bright field microscope (10×) and the wound spaces were measured using Olympus DP controller software. The percentage of change in the wound space was calculated using the following formula: % change = (average space at time 0 h) - (average space at time 24 h)/(average space at time 0 h) ×100. Relative cell migration was calculated by dividing the percentage change in the wound space of treated cells by that of the control cells in each experiment.

### Cell invasion assay

An invasion assay was performed using a trans-well Boyden chamber (8 μm pore size; BD Bioscience, MA, USA), as previously described [21]. Before the experiment, the trans-well membrane was coated with 0.5% matrigel overnight at 37 °C. The treated cells after 48 h were seeded at a density of 2 × 10^4^ cells/well in the upper chamber supplemented with serum free medium, while complete medium containing 10% FBS was added to the lower chamber compartment. After incubation at 37 °C, the non-invaded cells in the upper chamber were gently removed with a cotton swab, whereas the cells that had moved to the lower compartment of the membrane were fixed with cold absolute methanol for 10 min and stained with 10 μg/ml Hoechst 33342 for 10 min. Finally, these cells were visualized and captured using a fluorescence microscope (Olympus IX5; 20×). Relative invasion level was calculated by dividing the number of invade cell of the phoyunnanin E-treated cells by that of the non-treated cells in each experiment.

### Morphology evaluation assay

To characterize the effect of phoyunnanin E on cell morphology, as previously described [21], phoyunnanin E-treated H460, H292, A549 and HaCaT cells were fixed with 4% paraformaldehyde for 10 min after which the cell membranes were permeabilized by 0.1% Triton-X in PBS for 5 min. Next, the cells were rinsed with PBS and blocked for unspecific binding with 0.2% BSA in PBS for 30 min, incubated with a 1:100 dilution of phalloidin-rhodamine in PBS for 30 min, rinsed in PBS three times and mounted in 50% glycerol in PBS. Cell morphology changes were visualized and captured using a fluorescence microscope (Olympus IX5; 40×). The number of filopodia/cell was calculated using the following formula: (number of filopodia formed/number of cells). Relative number of filopodia/cell was calculated by dividing the number of filopodia/cell of the phoyunnanin E-treated cells by that of the non-treated cells in each experiment.

### Western blot analysis

After phoyunnanin E treatment, H460 cells were incubated with lysis buffer containing 20 mM Tris HCl (pH 7.5), 1% Triton X-100, 150 mM sodium chloride, 10% glycerol, 1 mM sodium orthovanadate, 50 mM sodium fluoride, 100 mM phenylmethylsulfonyl fluoride and protease inhibitor cocktail (Roche Molecular Biochemical) for 30 min on ice. The cellular lysates were collected and their protein content was determined using a BCA protein assay kit (Pierce Biotechnology, Rockford, IL, USA). Equal amounts of protein from each sample were separated by SDS-PAGE and transferred to 0.45 μm nitrocellulose membranes (Bio-Rad). The resulting blots were blocked for 1 h with 5% non-fat dry milk in TBST (Tris-buffer saline with 0.1% Tween containing 25 mM Tris-HCl (pH 7.5), 125 mM NaCl and 0.1% Tween 20) and incubated with the appropriate primary antibodies at 4 °C overnight. After three washes in TBST, the blots were incubated with horseradish peroxidase (HRP)-conjugated secondary antibodies for 2 h at room temperature. Finally, protein bands were detected using an enhancement chemiluminescence substrate (Supersignal West Pico; Pierce, Rockford, IL, USA) and quantified using the analyst/PC densitometry software package (Bio-Rad).

### Statistical analysis

Data from three or more independent experiments are presented as mean ± standard deviation (SD). Multiple comparisons for significant differences between multiple groups were performed using analysis of variance (ANOVA), followed by individual comparisons with Scheffe’s post-hoc test. Statistical significance was considered at *p* < 0.05.

## Results

### Effect of phoyunnanin E on cell viability and proliferation of human lung cancer H460 cells

Phoyunnanin E (Fig. 1a) was isolated from *D. venustum*., and the isolated sample with more than 95% purity was used in this study. Its purity was determined NMR spectroscopy. To determine the appropriate doses of phoyunnanin E to be used in the following experiments, we evaluated the viabilities of human lung cancer H460 cells treated with phoyunnanin E. To this end, the cells at 80–90% confluence were seeded, incubated for 10 h, and treated with various concentrations (0–100 μM) of phoyunnanin E for 24 h, after which cell viability was determined by MTT assay. Figure 1b shows that significant cytotoxic effects of phoyunnanin E could be found at the concentrations from 50 to 100 μM. To confirm whether the compound at concentrations from 0 to 20 μM could be considered non-cytotoxic to the cells, the occurrence of apoptotic and necrotic cells was determined by Hoechst33342/PI nuclear staining. Results indicated that neither apoptotic nor necrotic cells were detected in the cells treated with phoyunnanin E at 0–10 μM, whereas 20–100 μM phoyunnanin E induced a significant increase in apoptotic and necrotic cells, indicated by the cells exhibiting condensed and/or fragmented nuclei, and the cells stained with PI, respectively (Fig. 1c, e). For proliferation, the non-toxic concentrations of phoyunnanin E (0–10 μM) were used. Figure 1d shows that phoyunnanin E at the concentrations from 0 to 10 μM had not altered the proliferation rate of the cells at 24 h. Meanwhile, the proliferation rate of treated cells had significantly decreased in a dose-dependent manner at the concentration of 10 μM at 48 h.

### Phoyunnanin E attenuates anchorage-independent growth and migration of lung cancer H460 cells

**Fig. 2:**
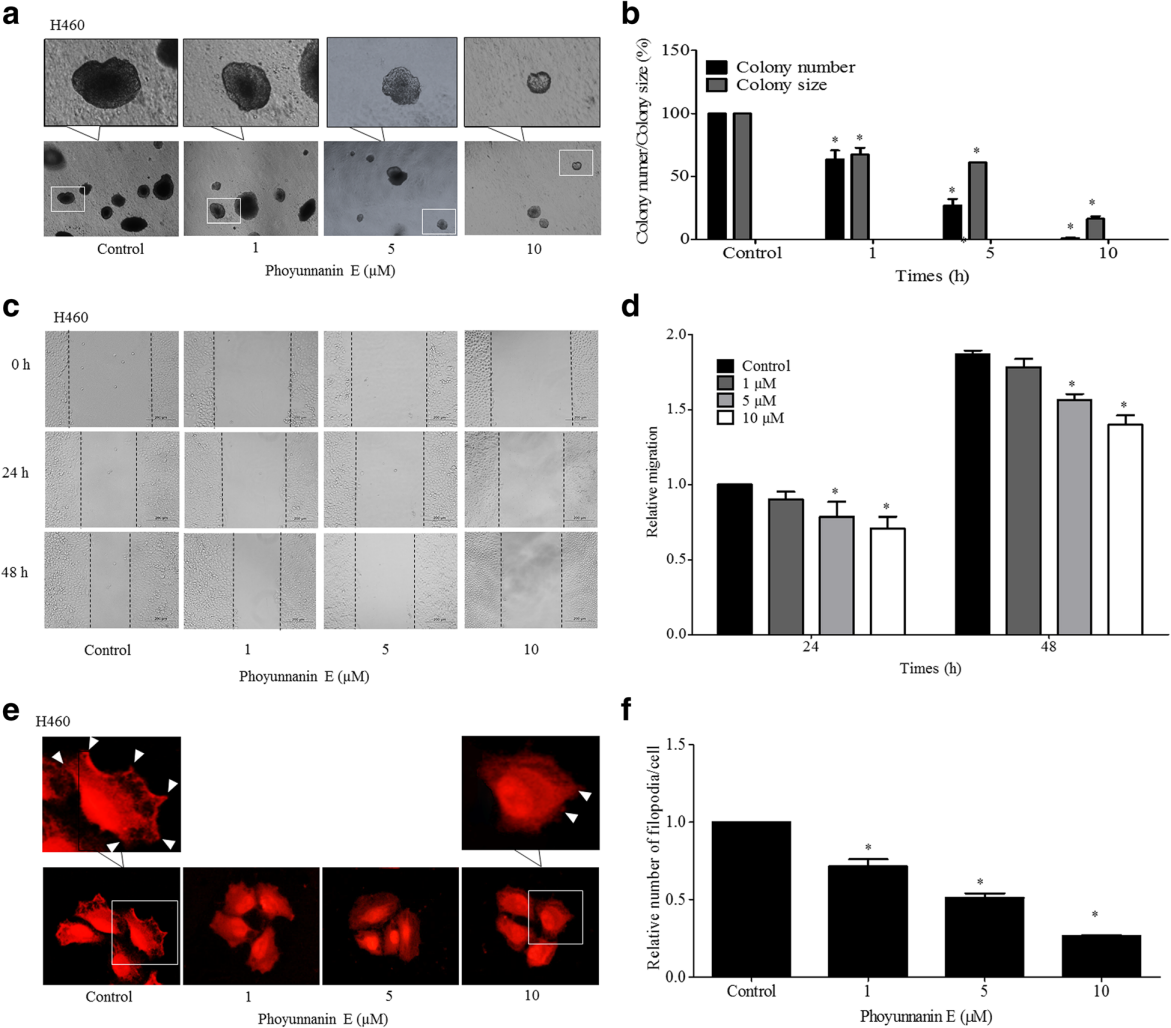
Phoyunnanin E suppresses anchorage-independent growth of H460 cells: H460 cells were treated with non-toxic doses of phoyunnanin E (0–10 μM) for 48 h. The treated cells were subjected to anchorage-independent growth assays for two weeks (**a**). The colony number as a percentage and the size of the treated cell were analyzed, and compared with the control (**b**). Phoyunnanin E decreased H460 cell migration: Cells were exposed to phoyunnanin E at the concentrations of 1, 5 and 10 μM, and migrations at 24 and 48 h were investigated. The migrating cells were captured (**c**). The relative cell migration was determined by comparing with the control (**d**). Effect of phoyunnanin E on filopodia formation: After treatment with non-toxic concentrations of phoyunnanin E for 48 h, cells were stained with phalloidin-rhodamine and examined using fluorescent microscopy. Filopodia characteristics are indicated by arrowheads (**e**). Relative number of filopodia per cell in H460 cells treated with phoyunnanin E, compared with the control (**f**). Data are shown as the mean ± SD (n = 3). * *P* < 0.05 versus non-treated control

The survival and growth of cancer cells in detached or anchorage-independent conditions have been linked with metastatic potential. We further evaluated the effect of phoyunnanin E on the growth of H460 cells in an anchorage-independent condition. Cells were treated with phoyunnanin E at non-toxic concentrations (0–10 μM) for 48 h, and single-cell suspensions of the treated and non-treated control cells were subjected to the anchorage-independent growth assay. The cells were allowed to grow for two weeks and the morphology of the cell colonies was captured and is presented in Fig. 2a. Results indicated that treatment of the cells with non-toxic concentrations of phoyunnanin E significantly decreased the formation of cancer cell colonies in terms of number and diameter of colonies, in a dose-dependent manner, in comparison to those of the control (Fig. 2b). Furthermore, wound healing assays was performed to determine the effect of phoyunnanin E on lung cancer cell migration. Cells were left untreated or pretreated with phoyunnanin E at non-toxic concentrations for 48 h, and then the cells were subjected to migration assays as described in Materials and Methods. Figure 2c and d show that phoyunnanin E significantly inhibited cell migration across the wound space at the concentrations of 5 and 10 μM, at 24 h and 48 h**,** compared to the non-treated control.

The cellular protrusion known as filopodia is an important indicator for motile cells. Filopodia formation has been observed in cancer cells during migration and invasion [23–25]. Therefore, we investigated the change in terms of filopodia formation in response to phoyunnanin E treatment. The cells were treated with various concentrations of phoyunnanin E (0–10 μM) for 48 h, after which the presence of filopodia was determined using a phalloidin-rhodamine staining assay. Figure 2e, and f show that cells treated with phoyunnanin E exhibited a significant decrease in filopodia numbers at the protrusion edges of the cells in a dose-dependent manner, when compared to their control.

### Phoyunnanin E decreases cancer cell migration in other human lung cancer cells

**Fig. 3:**
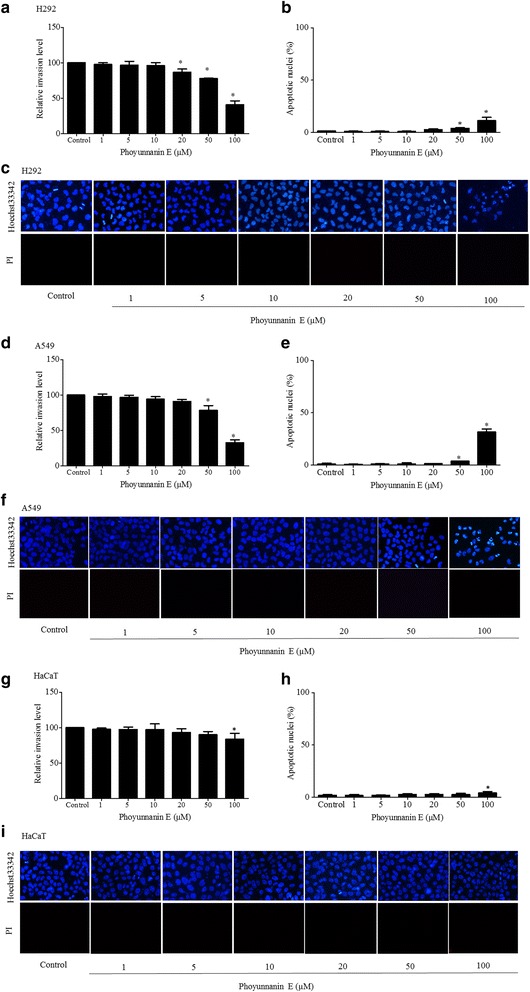
Viability of non-small cell lung cancer cells (H292, A549) and normal human keratinocyte (HaCaT) cells in response to various concentrations of phoyunnanin E (0–100 μM) treatment for 24 h (**a**, **d**, **g**). Cell viability was evaluated using 3-[4,5-dimethylthiazol-2-yl]-2,5 diphenyl tetrazoliumbromide (MTT) assays. Percentages of apoptotic in H292, A549 and HaCaT cells treated with phoyunnanin E (**b**, **e**, **h**). Apoptotic and necrotic cell death of H292, A549 and HaCaT cells after phoyunnanin E treatment, determined by Hoechst 33342/PI co-staining and visualized by fluorescence microscopy (**c**, **f**, **i**). Data are shown as the mean ± SD (*n* = 3). * *P* < 0.05 versus non-treated control

To evaluate the effect of phoyunnanin E on migratory activity, we first determined the viabilities of human lung cancer H292, A549 cells and HaCaT cells treated with various concentrations (0–100 μM) of phoyunnanin E. The results indicated that phoyunnanin E at the concentrations ranging from 1 to 10 μM, 1 to 20 μM and 1 to 50 μM have no significant cytotoxicity to H292, A549 and HaCaT cells, respectively. (Fig. 3a, d, g). To confirm the effect of phoyunnanin E on cells toxicity, the cells were treated with phoyunnanin E 24 h. Apoptotic and necrotic cells were determined by Hoechst33342/PI nuclear staining. Results indicated that phoyunnanin E at 0–100 μM have no effect on necrosis in H292, A549 and HaCaT cells. Apoptotic cells could not detect in H292 and A549 cells treated with phoyunnanin E at 0–20 μM, whereas 50–100 μM phoyunnanin E induced a significant increase in apoptotic cells, indicated by the cells exhibiting condensed and/or fragmented nuclei (Fig. 3b, c, e and f). In addition, phoyunnanin E at 0–50 μM has no effect on apoptosis in HaCaT cells (Fig. 3h and i).

**Fig. 4:**
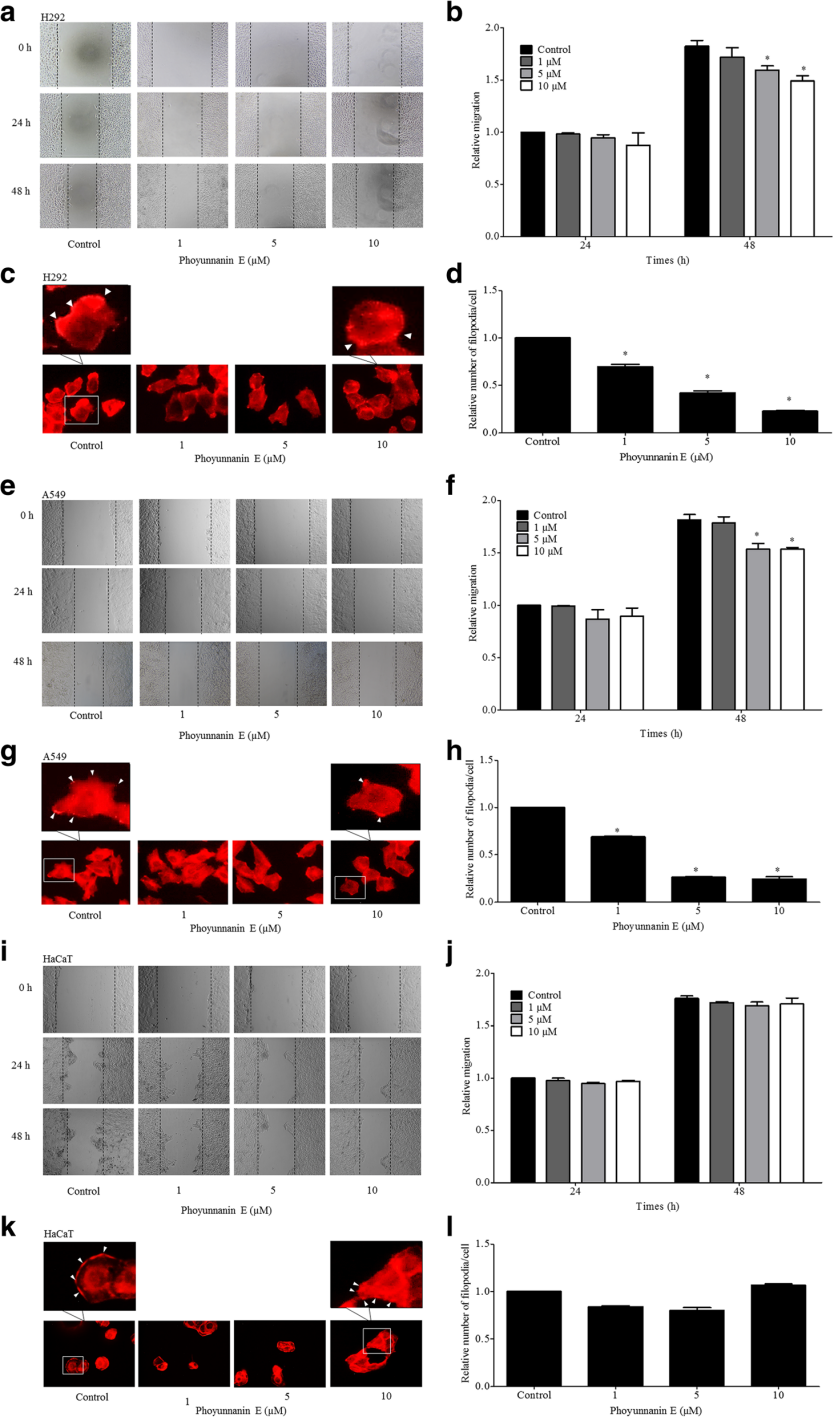
Phoyunnanin E decreases H292 and A549 cell migration: Cells were exposed to phoyunnanin E at concentrations of 1, 5 and 10 μM, and migrations at 24 and 48 h were investigated. The migrating cells were captured (**a**, **e**, and **i**). The relative cell migration was determined by comparing with the control (**b**, **f**, and **j**). Effect of phoyunnanin E on filopodia formation. After treating with non-toxic concentrations of phoyunnanin E for 48 h, cells were stained with phalloidin-rhodamine and examined using fluorescent microscopy. Filopodia characteristics are indicated by arrowheads (**c**, **g**, and **k**). Relative numbers of filopodia per cell in H292, A549, and HaCaT cells treated with phoyunnanin E compared with control (**d**, **h**, and **l**) are shown. Data are shown as mean ± SD (*n* = 3). * *P* < 0.05 versus non-treated control

In order to determine the anti-migration effect of the compounds, other human lung cancer cells (H292 and A549) and normal human keratinocytes were utilized. Cells were left untreated or treated with phoyunnanin E at non-cytotoxic concentrations (1–10 μM) for 48 h, and the cells were subjected to migration assays as described in Materials and Methods. Figure 4a and e show that phoyunnanin E had significantly inhibited migration of H292 and A549 human lung cancer cells at the concentrations of 5 and 10 μM at 48 h, compared to the non-treated control. Interestingly, our results showed that phoyunnanin E has no effect on the migration activity of HaCaT cells (Fig. 4i).

Furthermore, we investigated the filopodia formation of A549, H292, and HaCaT cells treated with phoyunnanin E. The cells were treated with various concentrations of phoyunnanin E (0–10 μM) for 48 h, after which the presence of filopodia was determined using a phalloidin-rhodamine staining assay. Figure 4c and g show that the lung cancer cells treated with phoyunnanin E exhibited a significant decrease in filopodia numbers at the protrusion edges of the cells in a dose-dependent manner, when compared to their control, while keratinocytes treated with phoyunnanin E exhibited only minimal change (Fig. 4k).

### Phoyunnanin E decreases cancer cell invasion

**Fig. 5:**
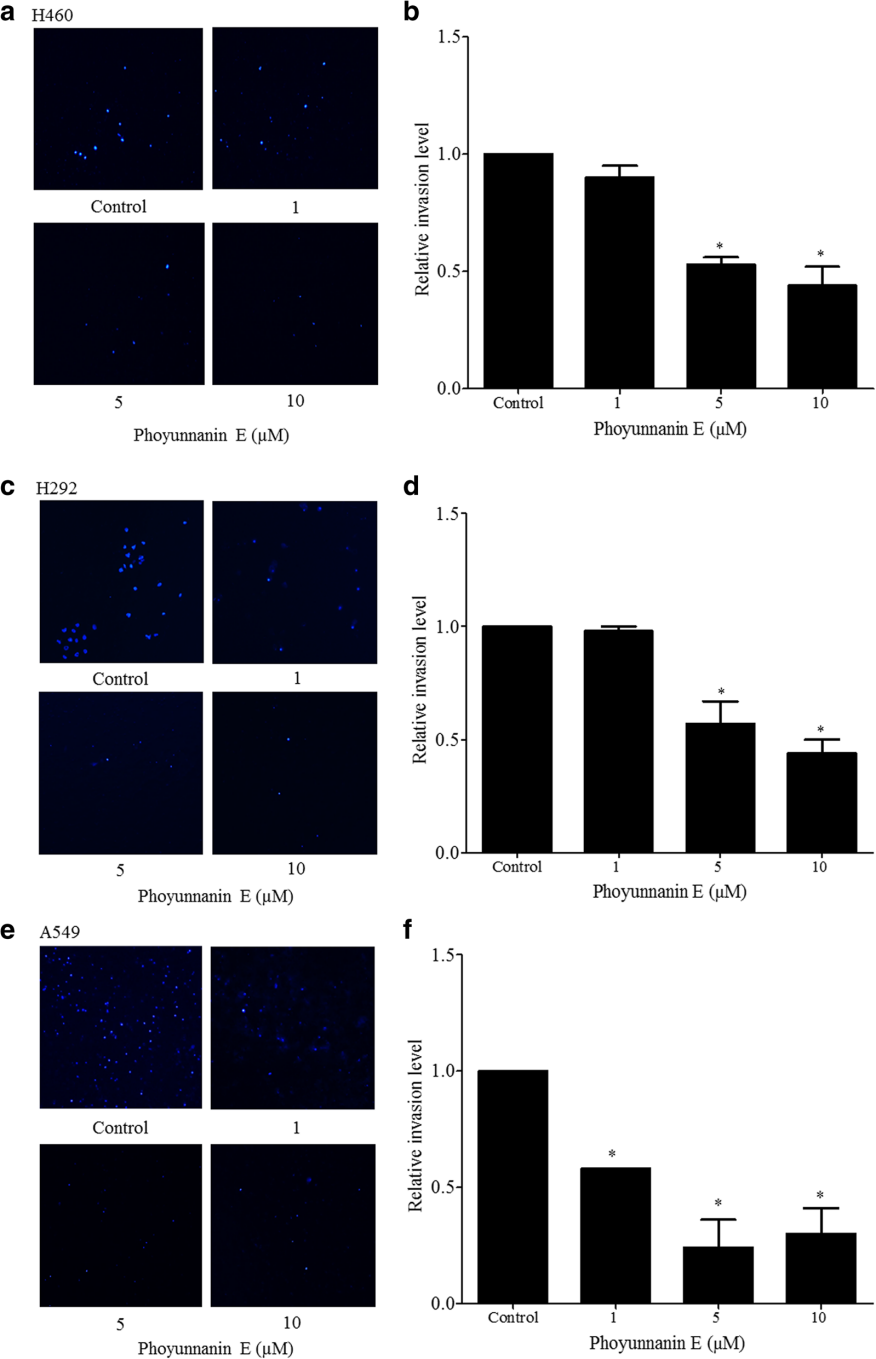
H460, H292 and A549 cell invasion was examined using transwell invasion assay. After 48 h, the invaded cells were stained with Hoechst 33342 and visualized by fluorescence microscopy (**a**, **c**, **e**). The relative invasion level was calculated as the number of invaded cells of the treatment groups divided by that of the untreated control group (**b**, **d**, **f**). Data are shown as the mean ± SD (*n* = 3). * *P* < 0.05 versus non-treated control

We further evaluated the anti-invasive effect of the compound in lung cancer cells. Human lung cancer cells (H460, H292 and A549) were left untreated or treated with phoyunnanin E at non-cytotoxic concentrations (1–10 μM) for 48 h, and the cells were subjected to invasion assays as described in Materials and Methods. Results indicated that phoyunnanin E were able to significantly decrease the number of cells invading across the matrix and transwell filter within 24 h in a dose-dependent manner, in comparison to those of their non-treated control (Fig. 5).

### Phoyunnanin E suppresses epithelial–mesenchymal transition (EMT)

**Fig. 6:**
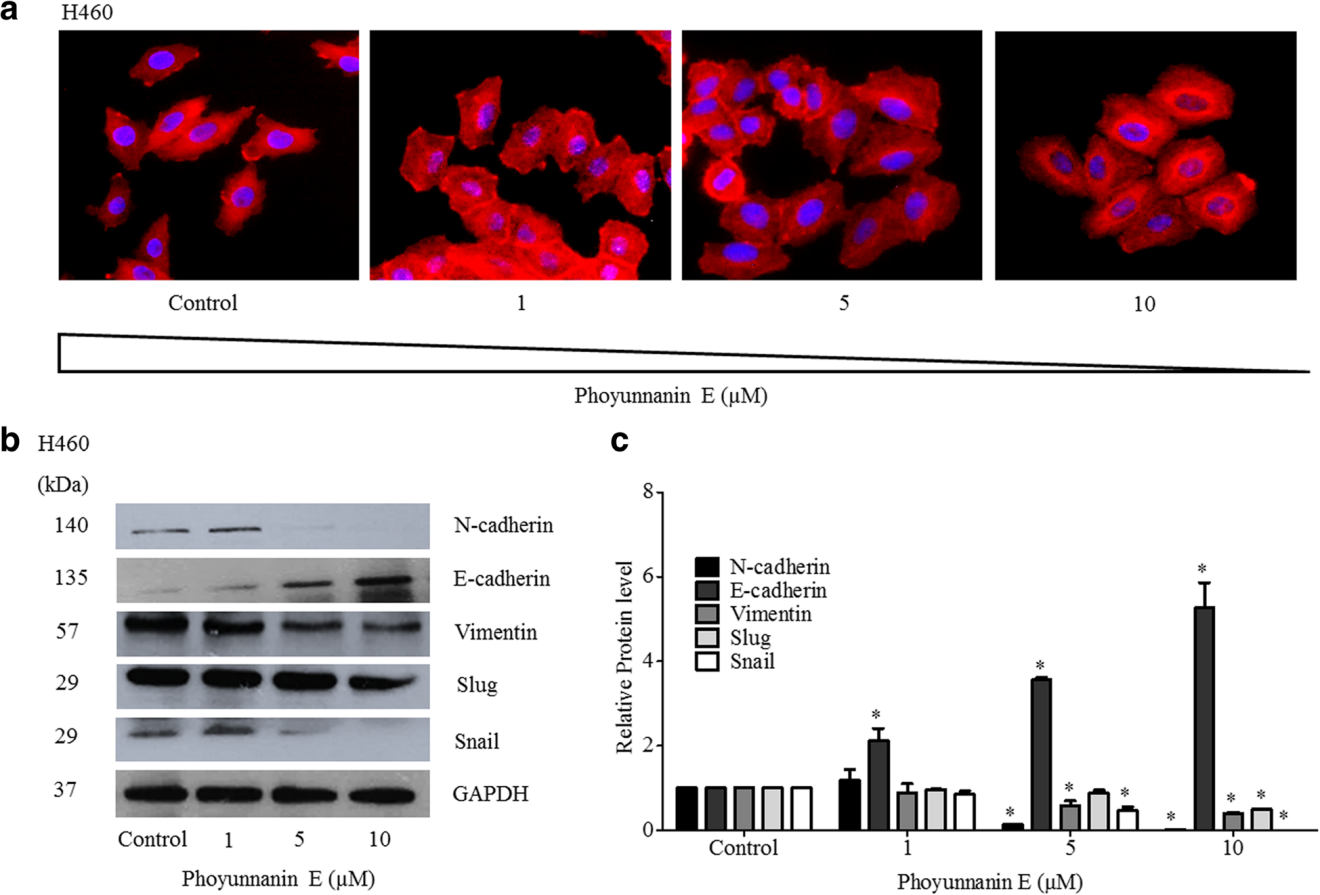
Effect of phoyunnanin E on epithelial to mesenchymal transition (EMT): Cells were treated with various concentrations of phoyunnanin E (0–10 μM) for 48 h. Cell morphology was examined under a fluorescence microscope (**a**). Cells treated with phoyunnanin E were subjected to western blotting, and the expression levels of N-cadherin, E-cadherin, vimentin, slug and snail were determined. To confirm equal loading of the samples, the blots were reprobed with a GAPDH antibody (**b**). The immunoblot signals were quantified by densitometry (**c**). The data are shown as mean ± SD (*n* = 3). *P < 0.05 versus non-treated control

The process of cancer cell transition from an epithelial-adhered phenotype to a mesenchymal phenotype has been shown to potentiate migration, invasion, anoikis resistance, and metastasis of cancers [6, 26, 27]. The key hallmarks of EMT are the alteration of cell morphology to spindle-like cells and alteration of proteins (including the E-cadherin to N-cadherin switch; and increase of vimentin, snail, and slug) [27–29]. To support the above finding of anchorage-independent growth of cancer cells, we next investigated EMT in the lung cancer cells treated with phoyunnanin E. Cells were treated with phoyunnanin E at non-cytotoxic concentrations for 48 h. The morphology of the cells was determined and is shown in Fig. 6a. In the presence of phoyunnanin E, the mesenchymal-like cells with elongated fibroblast-like morphology were converted into epithelial cells. Western-bolt protein analysis revealed that cells treated with the indicated concentrations of phoyunnanin E exhibited reversal of the E-cadherin to N-cadherin. The treated cells showed a significant decrease in levels of N-cadherin, but the increase of E-cadherin can be compared to non-treated control cells (Fig. 6b and c). In addition, phoyunnanin E reduced the EMT-related proteins of vimentin, snail, and slug; however, we found a change in slug level only at 10 μM (Fig. 6b and c).

### Phoyunnanin E down-regulates integrins αv and β3 and the regulatory proteins in cell migration

**Fig. 7:**
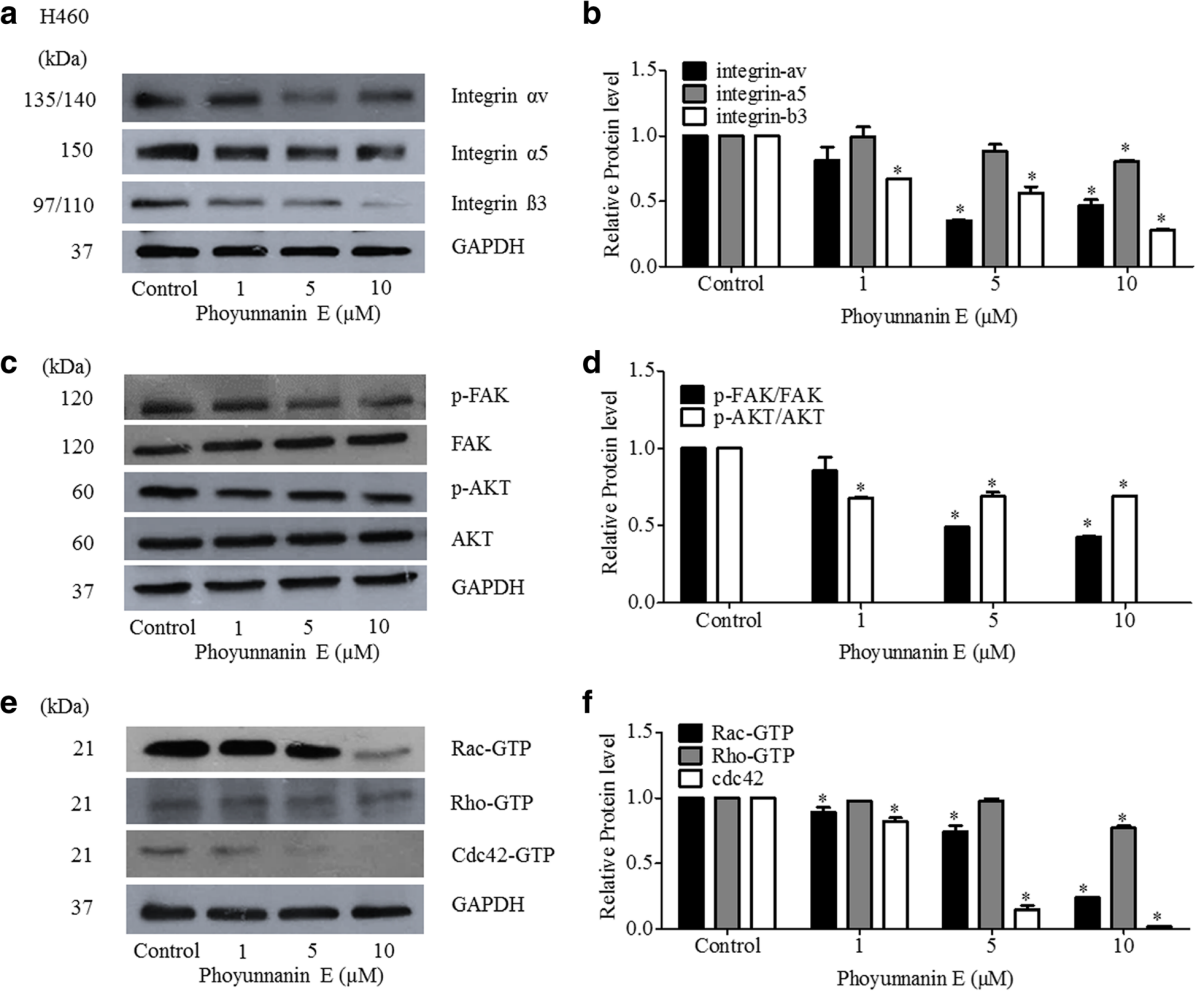
Phoyunnanin E decreases cell migration through integrin downregulation. Cells treated with phoyunnanin E were subjected to western blotting after 48 h, and the expression levels of integrins αv, α5, and β3; phosphorylated Akt (S 473); total Akt, phosphorylated FAK (Y 397); and total FAK were determined. To confirm equal loading of the samples, the blots were reprobed with a GAPDH antibody (**a**, **c** and **e**). The immunoblot signals were quantified by densitometry (**b**, **d** and **f**). The data are shown as mean ± SD (*n* = 3). *P < 0.05 versus non-treated control

Certain integrins, including integrins αv and β3, have been shown to be strongly linked with the aggressive and invasive phenotypes of cancers [11–13]. We tested whether the compound could attenuate the expression of these metastasis-associated integrins. The levels of integrins αv, α5, and β3 were evaluated by western blot analysis in cells treated with phoyunnanin E at non-toxic concentrations. We found that phoyunnanin E treatment significantly decreased the expression of integrins αv and β3 in H460 cells, in a dose-dependent manner, whereas it slightly decreased the expression of integrin α5 (Fig. 7a, b). Phoyunnanin E had no effect on integrin β1 (data not shown). Therefore, phoyunnanin E inhibits the migration of NSCLC cells, at least in part, by decreasing the expression of integrins αv and β3.

To further confirm the effect of phoyunnanin E on cancer cell migration, the key regulatory proteins for cancer cell migration were monitored in the cells treated with such a compound. Total FAK, the active (phosphorylated at Try397) form of FAK (p-FAK (Try397)); total AKT; active (phosphorylated at Ser473) AKT (p-AKT (Ser473)); Cdc42; active Rac1 (Rac-GTP); and active RhoA (Rho-GTP) were determined in the cells treated with phoyunnanin E. Protein analysis revealed that phoyunnanin E treatment significantly suppressed the levels of p-FAK, p-AKT and their downstream effectors, including Rac-GTP and Cdc42; however, it only slightly decreased the expression of Rho-GTP (Fig. 7c-f). Taken together, we have reported the anti-migration and EMT-suppressive activities of phoyunnanin E, and the underlying mechanisms of action covered by the reduction of EMT regulatory proteins, migratory-associated integrins αv and β3, and active FAK and AKT, which conferred the decrease of functions to their downstream migratory proteins.

## Discussion

As lung cancer is the major cause of cancer-related death worldwide and metastasis in this cancer has been shown to be a critical factor in determining patient survival [30, 31], novel efficient therapies as well as effective therapeutic agents that can overcome metastasis are considered very interesting. Cancer metastasis is the multistep process of cancer cells spreading from their original site to other distant sites of the body [32–34]. During metastasis, cancer cells in an anchorage-independent condition are likely to die in the process of anoikis, a detachment-induced apoptosis; however, metastatic cells utilize several mechanisms to escape anoikis and consequently grow at distant sites [22, 35, 36]. Among several potentiating factors of metastasis, the transition of epithelial to mesenchymal phenotype, known as EMT [6, 37, 38], has garnered most attention [39–41]. EMT has been shown to facilitate cancer progression and aggressive behaviors of cancer cells, such as increased cell migration and invasion, and anoikis resistance [42–46]. Here, we found that phoyunnanin E, a pure compound isolated from *D. venustum* and related species, has anti-migration and EMT-suppression activities. In the cell viability test, the concentrations that allow over 90% of cells to survive were used for investigating EMT inhibition based on anchorage-independent growth and anti-migration. Phoyunnanin E was shown to decrease EMT, indicated by the decrease of EMT markers such as N-cadherin, vimentin, snail, and slug, together with the increase in the epithelial marker, E-cadherin (Fig. 6b, c). Previous studies indicated that EMT is a highly complex process requiring extensive changes in adhesion, cell morphology and protein expression [4, 47, 48]. The regulation of the EMT process has various mechanisms for activation. It has cross-talk between signaling molecules leading to complex biochemical circuits. Down regulation of E-cadherin and up regulation of N-cadherin are the major molecular events regarding the reduction in cell-cell adhesion and facilitation of cell movement [49, 50]. In addition, vimentin is an important protein regulator of EMT and the down-regulation of vimentin has been shown to decrease cell migration and transcription factors of EMT, such as snail and slug [51–53]. In this study, we found that phoyunnanin E could reverse the E-cadherin to N-cadherin switch and reduce vimentin and the related EMT transcription factors, snail and slug (Fig. 6b, c).

Lung cancer cells bind with the extracellular matrix and lead to integrin clustering at the adhesion, and subsequent recruitment and activation, of signaling proteins such as FAK, PI3K/AKT, and the Rac-Rho pathway [17, 21, 54]. Our results suggest that decreases in the expression of integrins αv and β3; p-FAK and the downstream signaling pathways (p-AKT, Rac1, Cdc42 and RhoA) inhibit the migratory activity of H460 cells (Fig. 7). These results fit with the previous studies indicating that not only does integrin regulate the cellular processes of survival, proliferation, and adhesion, but it also regulates cell motility [55–57]. Integrin initiates transmembrane signaling by activating FAK through phosphorylation at Tyr 397 for cell migration [58–60]. The p-FAK activates downstream effectors such as AKT and Rho families. In particular, the phosphorylation of AKT at Ser473 was shown to be critical for cancer cell migration [61–63].

The Cdc42-GTP downstream migratory protein regulates cell migration by stimulating the formation of cell-membrane protrusion filopodia at the edge of the migrating side [21, 64–66]. We found that the decrease of Cdc42 in response to phoyunnanin E treatment correlated with the reduction of cellular filopodia (Fig. 2e, f and 7).

Mounting evidence has demonstrated that several natural products have abilities to suppress EMT in lung cancer. Previously, a bibenzyl 4,5,4′-trihydroxy-3,3′-dimethoxybibenzyl [TDB], isolated from *Dendrobium ellipsophyllum*, was shown to suppress EMT and sensitize lung cancer cells to anoikis [67]. Gigantol, a pure compound isolated from *Dendrobium draconis,* has been shown to suppress EMT in lung cancer cells [68, 69]. In addition, bibenzyl compounds from *Dendrobium pulchellum*, including moscatilin, chrysotobibenzyl, chrysotoxine and crepidatin, have been demonstrated to suppress EMT in a lung cancer cell model, and such suppression resulted in the induction of anoikis and decreased growth in an anchorage-independent manner [70, 71].

## Conclusion

**Fig. 8:**
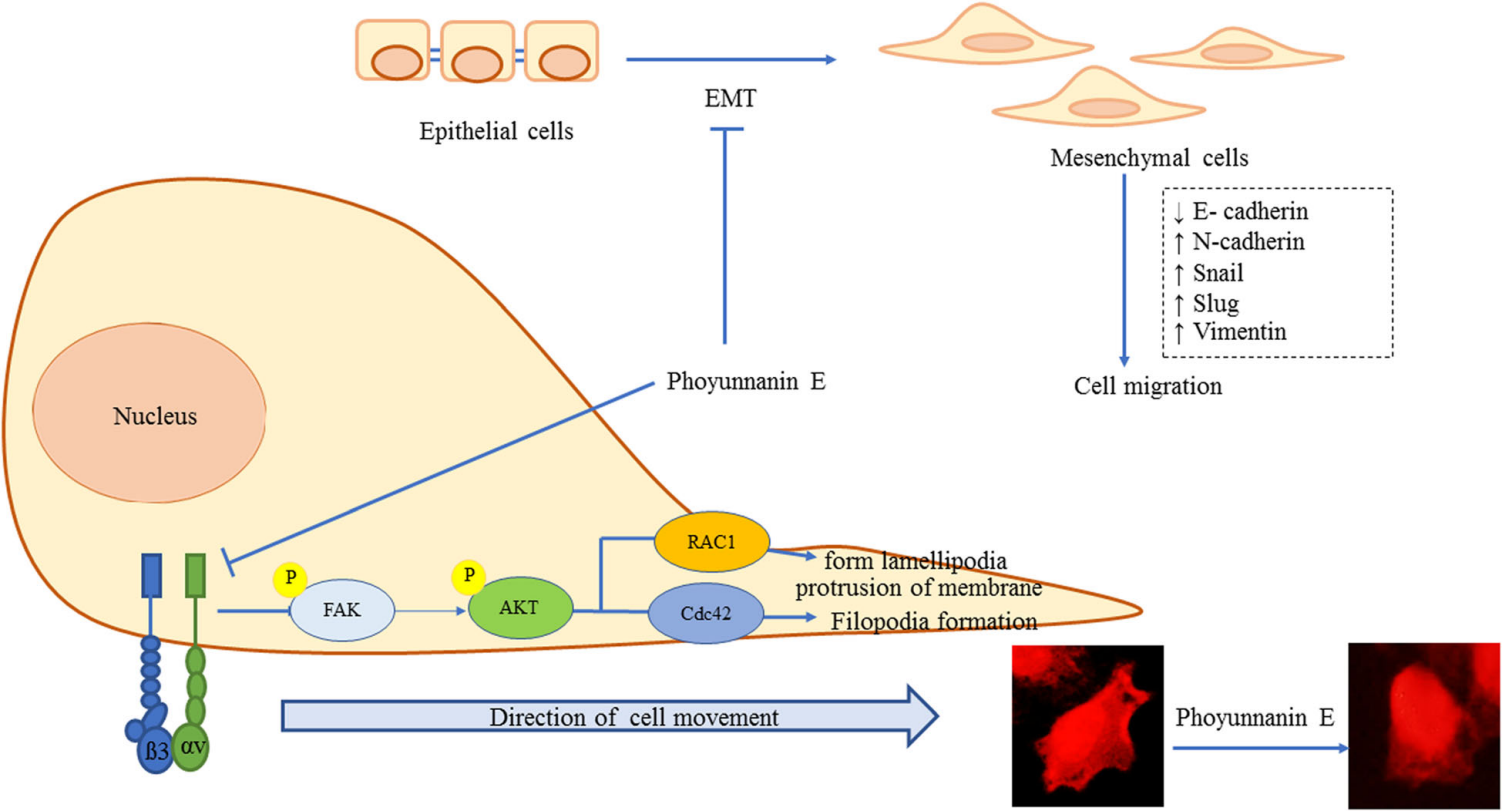
Schematic mechanism of phoyunnanin E in attenuating lung cancer cell migration. Phoyunnanin E suppresses cell migration via integrin downregulation and decreased EMT in lung cancer cells

In conclusion, we show here for the first time that phoyunnanin E inhibits migration and growth in an anchorage-independent manner with detailed mechanisms of action covering EMT suppression, reduction of migratory-associated integrins αv and β3, and suppression of FAK/AKT signals which consequently suppressed downstream migratory proteins as concluded in the summarized schematic figure (Fig. 8). This novel data could support the development of phoyunnanin E for anti-metastasis in lung cancer.

## Abbreviations

AKT: Protein Kinase B
BCA: Bicinchoninic acid
BSA: Bovine serum albumin
Cdc42: Cell division cycle 42
DMEM: Dulbecco’s Modified Eagle’s Medium
DMSO: Dimethyl sulfoxide
EMT: Epithelial to mesenchymal transition
EtOAc: Ethyl acetate
FAK: Focal Adhesion Kinase
FBS: Fetal bovine serum
GAPDH: Glyceraldehyde 3-phosphate dehydrogenase
HaCaT: Human keratinocyte
HCl: Hydrochloric acid
HRP: Horseradish peroxidase
MeOH: Methanol
MTT: 3-(4,5-dimethylthiazol-2-yl)-2,5-diphenyltetrazoliumbromide
NMR: Nuclear magnetic resonance
NSCLC: Non-small cell lung cancer
OD: Optical densities
p-AKT: Phosphorylation of Protein Kinase B
PBS: Phosphate buffer saline
p-FAK: Phosphorylation of Focal Adhesion Kinase
PI: Propidium iodide
Rac1: Ras-related C3 botulinum toxin substrate 1
RhoA: Ras homolog gene family, member A
RPMI: Roswell Park Memorial Institute 1640 medium
SDS-PAGE: Sodium dodecyl sulfate polyacrylamide gel electrophoresis
TBST: Tris-buffer saline with 0.1% Tween containing 25 mM Tris-HCl (pH 7.5), 125 mM NaCl and 0.1% Tween 20
