# Correction: Phoyunnanin E inhibits migration of non-small cell lung cancer cells via suppression of epithelial-to-mesenchymal transition and integrin αv and integrin β3

**DOI:** 10.1186/s12906-023-04034-4

**Published:** 2023-06-15

**Authors:** Nareerat Petpiroon, Boonchoo Sritularak, Pithi Chanvorachote

**Affiliations:** 1 Cell-Based Drug and Health Product Development Research Unit, Faculty of Pharmaceutical Sciences, Chulalongkorn University, Bangkok, 10330 Thailand; 2 Department of Pharmacology and Physiology, Faculty of Pharmaceutical Sciences, Chulalongkorn University, Bangkok, 10330 Thailand; 3 Department of Pharmacognosy and Pharmaceutical Botany, Faculty of Pharmaceutical Sciences, Chulalongkorn University, Bangkok, Thailand

**Correction: BMC Complement Med Ther 17, 553 (2017)**

**https://doi.org/10.1186/s12906-017-2059-7**

Following publication of the original article [1], the authors identified errors in Fig. 4. The correct figure is given below.

**Fig. 4:**
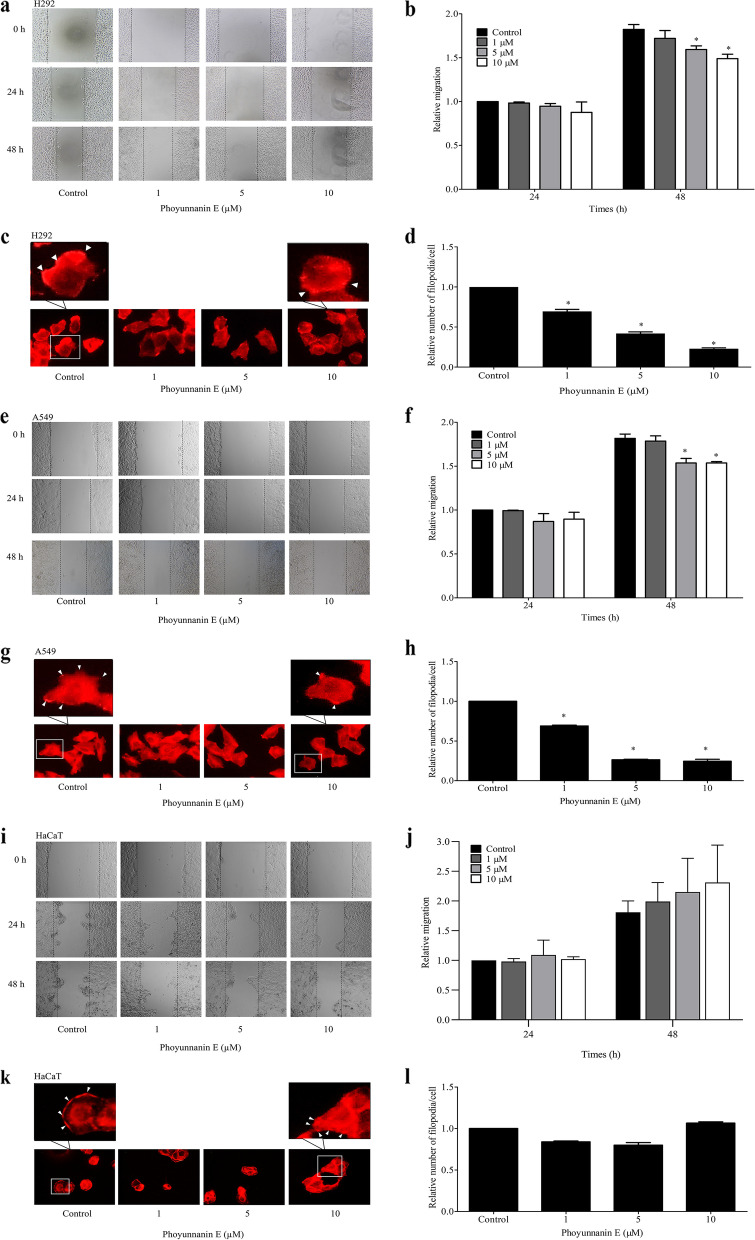
Phoyunnanin E decreases H292 and A549 cell migration: Cells were exposed to phoyunnanin E at concentrations of 1, 5 and 10 μM, and migrations at 24 and 48 h were investigated. The migrating cells were captured (**a**, **e**, and **i**). The relative cell migration was determined by comparing with the control (**b**, **f**, and **j**). Effect of phoyunnanin E on filopodia formation. After treating with non-toxic concentrations of phoyunnanin E for 48 h, cells were stained with phalloidin-rhodamine and examined using fluorescent microscopy. Filopodia characteristics are indicated by arrowheads (**c**, **g**, and **k**). Relative numbers of filopodia per cell in H292, A549, and HaCaT cells treated with phoyunnanin E compared with control (**d**, **h**, and **l**) are shown. Data are shown as mean ± SD (*n* = 3). * *P* < 0.05 versus non-treated control

The original article has been corrected.
